# BISCIT: Biliary interventions in critically ill patients with secondary sclerosing cholangitis—a study protocol for a multicenter, randomized, controlled parallel group trial

**DOI:** 10.1186/s13063-023-07260-w

**Published:** 2023-03-31

**Authors:** Klaus Stahl, Friederike Klein, Torsten Voigtländer, Anika Großhennig, Thorsten Book, Tobias Müller, Alexander Wree, Armin Kuellmer, Jochen Weigt, Alexander Dechene, Edris Wedi, Arne Kandulski, Christian M. Lange, Dennis Holzwart, Dorothee von Witzendorff, Kristina I. Ringe, Heiner Wedemeyer, Benjamin Heidrich, for the BISCIT Study group, Paul Schirmer, Henrike Lenzen, Ute Denkena, Christoph Schindler, Christian M. Lange

**Affiliations:** 1 Hannover Medical School, Department of Gastroenterology, Hepatology and Endocrinology, Carl-Neuberg Strasse 1, 30625 Hannover, Germany; 2 Department of Gastroenterology, Clementinenkrankenhaus Hannover, Hannover, Germany; 3 Department of Biostatistics, Hannover Medical School, Hannover, Germany; 4 Department of Hepatology and Gastroenterology, Charite University Medicine Berlin, Campus Charite Mitte/Campus Virchow Clinic, Berlin, Germany; 5 Clinic for Gastroenterology, Hepatology, Endocrinology and Infectious Diseases, University Hospital Freiburg, Freiburg, Germany; 6 Clinic for Gastroenterology, Hepatology and Infectious Diseases, University Hospital Magdeburg, Magdeburg, Germany; 7 Department of Gastroenterology and Endocrinology, Hospital Nurnberg, Nurnberg, Germany; 8 Department of Gastroenterology, Gastro-Oncology and Interventional Endoscopy, Sana Hospital Offenbach, Offenbach, Germany; 9 Department of Gastroenterology, Endocrinology, Infectious Diseases and Rheumatology, University Hospital Regensburg, Regensburg, Germany; 10 Department of Gastroenterology and Hepatology, University Hospital Munich (LMU), Munich, Germany; 11 Department of Diagnostic and Interventional Radiology, Hannover Medical School, Hannover, Germany; 12 Zentrum Klinische Studien (ZKS), Hannover Medical School, Hannover, Germany

**Keywords:** Secondary sclerosing cholangitis, Critical ill patients, Biliary interventions, Randomized-controlled trial, Cholangiosepsis, Endoscopic retrograde cholangiography

## Abstract

**Background:**

Progress of cholangitis to cholangiosepsis is a frequent observation in patients with secondary sclerosing cholangitis in critically ill patients (SSC–CIP). Adequate biliary drainage may reduce episodes of cholangiosepsis and therefore stabilize liver function and improve survival. The primary objective of the BISCIT study is to demonstrate that scheduled biliary interventions will reduce incidence of cholangiosepsis, liver transplantation, or death in patients with SSC–CIP.

**Methods:**

A total of 104 patients will be randomized at ten study sites. Patients with SSC–CIP, confirmed by endoscopic retrograde cholangiography (ERC), will be randomized 1:1 either in the intervention group which will be treated with scheduled biliary interventions (i.e., therapeutic ERC) every 8 weeks for 6 months or in the control group which will receive standard of care. The randomization will be stratified by center. The composite primary efficacy endpoint is defined as (1) occurrence of death, (2) necessity of liver transplantation, or (3) occurrence of cholangiosepsis within 6 months following randomization.

**Discussion:**

Prospective evaluation of endoscopic treatment procedures is urgently needed to establish an evidence-based therapeutic treatment algorithm in SSC–CIP. A positive trial result could change the current standard of care for patients with SSC–CIP. The results of this study will be disseminated through presentations at international congresses, workshops, and peer-reviewed publications.

**Trial registration:**

The trial was registered at ClinicalTrials.gov (NCT05396755, date of registration: May 31, 2022, last update: May 31, 2022).

## Administrative information

**Table:** 

Title	BISCIT: Biliary Interventions in critically ill patients with Secondary Sclerosing Cholangitis – a multicenter, randomized, controlled parallel group trial
Trial Registration	ClinicalTrials.gov, NCT05396755, date of registration: May 31^st^ 2022
Protocol Version	Version 1.0, February 23^rd^ 2022
Funding	German Research Foundation (DFG, VO 2458/1–1) The German Research Foundation is neither involved in developing the design of the study nor in collection, analysis, and interpretation of the data or in writing of the manuscript.
Author Details	Klaus Stahl^1^, Friederike Klein^1^, Torsten Voigtländer^2^, Anika Großhennig^3^, Thorsten Book^2^, Tobias Müller^4^, Armin Kuellmer^5^, Jochen Weigt^6^, Alexander Dechene^7^, Edris Wedi^8^, Arne Kandulski^9^, Christian M. Lange^10^, Dennis Holzwart^3^, Dorothee von Witzendorff^1^, Kristina I. Ringe^11^, Heiner Wedemeyer^1^, Benjamin Heidrich^1^ ^1^Hannover Medical School, Department of Gastroenterology, Hepatology and Endocrinology, Hannover, Germany ^2^Clementinenkrankenhaus Hannover, Department of Gastroenterology, Hannover, Germany ^3^Hannover Medical School, Department of Biostatistics, Hannover, Germany ^4^Charite University Medicine Berlin, Campus Charite Mitte/Campus Virchow Clinic, Department of Hepatology and Gastroenterology, Berlin, Germany ^5^University Hospital Freiburg, Clinic for Gastroenterology, Hepatology, Endocrinology and Infectious Diseases, Freiburg, Germany ^6^University Hospital Magdeburg, Clinic for Gastroenterology, Hepatology and Infectious Diseases, Magdeburg, Germany ^7^Hospital Nurnberg, Department of Gastroenterology and Endocrinology, Nurnberg, Germany ^8^Sana Hospital Offenbach, Department of Gastroenterology, Gastro-oncology and Interventional Endoscopy, Offenbach, Germany ^9^University Hospital Regensburg, Department of Gastroenterology, Endocrinology, Infectious Diseases and Rheumatology, Regensburg, Germany ^10^University Hospital Munich (LMU), Department of Gastroenterology and Hepatology, Munich, Germany ^11^Hannover Medical School, Department of Diagnostic and Interventional Radiology, Hannover, Germany
Name and Contact Information for Trial Sponsor	Hannover Zentrum für Klinische Studie (ZKS), Hannover Medical School Carl-Neuberg Strasse 1, 30,625 Hannover, Germany
Role of Sponsor	Investigator initiated trial

## Trial status

The current version of the study protocol described in the manuscript is Version 1.0, February 23, 2022. The trial was registered at ClinicalTrials.gov (NCT05396755, date of registration: May 31, 2022, last update: May 31, 2022). No patient has yet been recruited. Approximate end of recruitment will be May 2025.

## Introduction

### Background and rationale

Secondary sclerosing cholangitis (SSC) is a condition characterized by progressive destruction of the biliary tree caused by a wide variety of stimuli including but not limited to toxic and infectious agents, immune-mediated mechanisms, and ischemia [1]. Recently, a new sub-entity of SSC has been defined in critically ill patients (SSC–CIP). SSC–CIP occurs weeks or months after the onset of a very broad range of critical conditions that include but are not limited to major trauma, burns, major thoracic or abdominal surgery, sepsis/septic shock, and the acute respiratory distress syndrome (ARDS) [2–5]. SSC–CIP frequently leads to recurrent acute cholangitis and cholangiosepsis, cirrhosis, hepatic decompensation, and ultimately death. Prognosis of SSC–CIP is dismal with an estimated survival of less than 50% of patients who develop SSC–CIP within 3–6 months [2–4]. Other publications estimate a 1-year survival of 55% and only 14% after 6 years [5]. Current understanding and management of SSC–CIP are based on a limited number of reports with small patient numbers [2–5]. The exact pathological mechanisms and clinical risk factors have not been elucidated conclusively. A number of trigger factors have been proposed as causes for the development of SSC–CIP, including prolonged hypotension, vasopressors administration, intensive mechanical ventilation, and prone positioning [6].

Therapeutic approaches for patients with SSC–CIP include endoscopic interventions via endoscopic retrograde cholangiography (ERC) with sphincterotomy, removal of biliary casts, flushing of the bile ducts, dilation therapy, stent placement, and placement of nasobiliary drainages [2–5]. Patients with clinical evidence of bacterial cholangitis are treated with broad spectrum antibiotics according to microbiologic testing if available [5]. Ursodeoxycholic acid (UDCA) administration has been suggested, although data on effectiveness are lacking [7]. In case of end-stage disease, liver transplantation is a therapeutic option in selected patients [8].

To date, efficacy of endoscopic interventions has not been proven and an evidence-based standard therapy for SSC–CIP remains to be defined. Programmed (e.g., repeated) ERC interventions are intended to improve biliary drainage, reduce episodes of cholangitis, prevent biliary cirrhosis, and preserve liver function. However, ERC interventions including sphincterotomy and insertion of different catheters and guide wires into the biliary system might on the contrary even be harmful by promoting bacterial cholangitis, hence further accelerating disease progression.

### Objectives

The objective of this trial is to clarify whether endoscopic interventions in patients with SSC–CIP are beneficial or if an alternative, then conservative strategy, should be rather preferred. Consequently, our results will have a strong impact on the clinical management of patients with SSC–CIP.

### Trial design

**Fig. 1:**
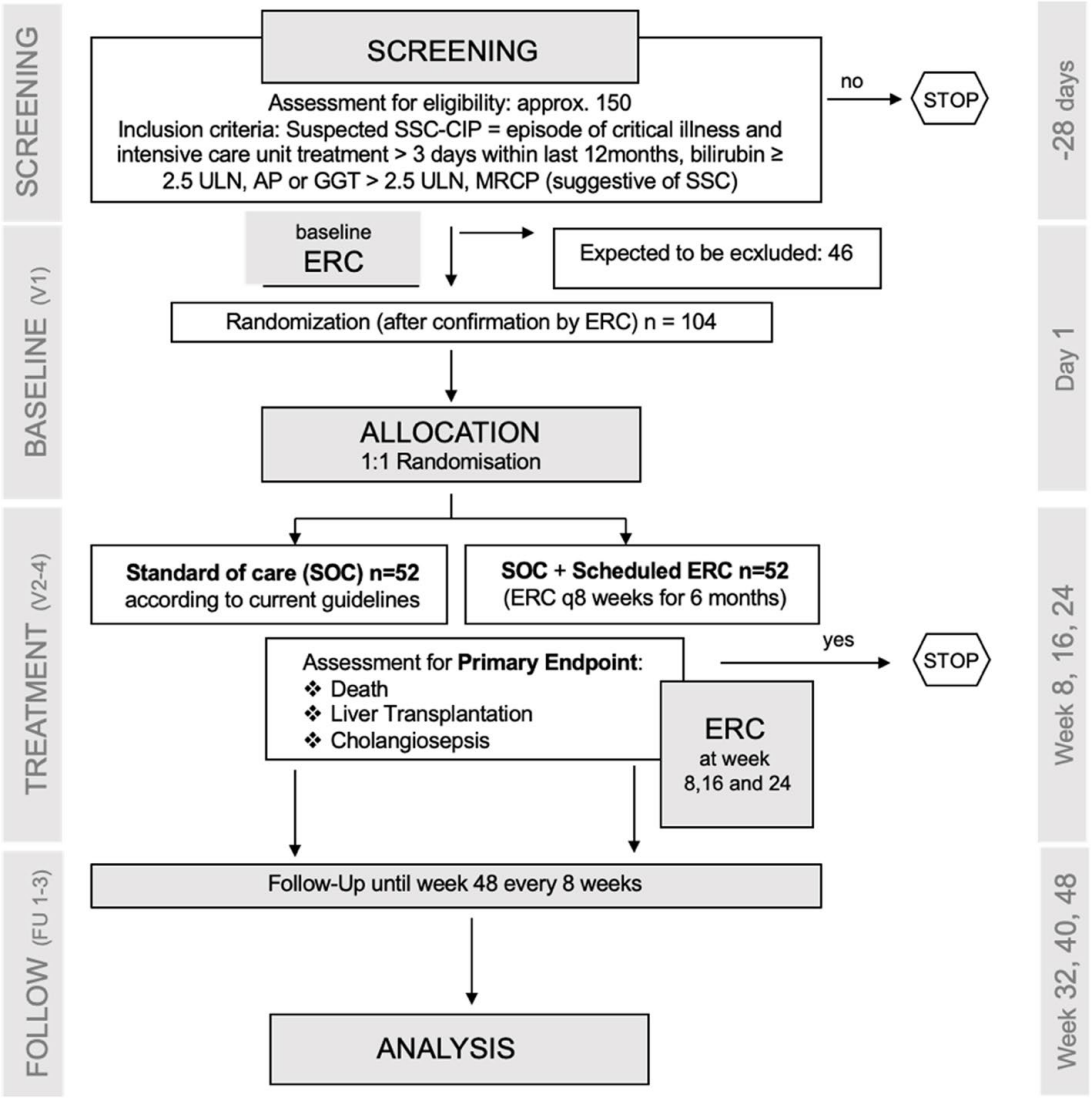
Trial structure

This is a multicenter, randomized, controlled parallel group trial. Patients with confirmed SSC–CIP will be randomized 1:1 either in the intervention group which will undergo scheduled invasive evaluation of the biliary tract with ERC with biliary interventions (i.e., therapeutic ERC) every 8 weeks for 6 months or in the control group which will receive non-interventional standard of care (SOC) (Fig. 1). The primary endpoint is a composite endpoint consisting of the individual components cholangiosepsis, liver transplantation, and death (whatever occurs first). Study participants will be followed up to 12 months following study inclusion. This study is an investigator-initiated trial funded by the German Research Foundation (DFG, VO 2458/1–1).

## Methods

### Participants, interventions, and outcomes

#### Study setting

A total of 104 patients with SSC–CIP (1:1 randomization) will be included in up to ten study centers in Germany. The study is currently planned at the following centers in Germany (BISCIT study group): Hannover Medical School, Department of Gastroenterology, Hepatology and Endocrinology; Charite University Medicine Berlin, Clinic for Gastroenterology and Hepatology; University Hospital Freiburg, Clinic for Gastroenterology, Hepatology, Endocrinology and Infectious Diseases; University Hospital Magdeburg, Clinic for Gastroenterology, Hepatology and Infectious Diseases, Magdeburg, Germany; Hospital Nuernberg, Department of Gastroenterology and Endocrinology; Sana Hospital Offenbach, Department of Gastroenterology, Gastro-oncology and Interventional Endoscopy, Offenbach, Germany; University Hospital Regensburg, Department of Gastroenterology, Endocrinology, Infectious Diseases and Rheumatology; University Hospital Munich (LMU), Department of Gastroenterology and Hepatology.

#### Eligibility criteria

Patients have to fulfill all of the following inclusion criteria to be eligible for participation in the study:

1. Men, women*, inter/divers, age ≥ 18 and ≤ 80 years (conscious or unconscious patients may be included)
2. Signed written informed consent obtained by patient or legal representative in case of unconscious patient
3. Willingness to comply with treatment and follow-up procedures
4. Suspected SSC–CIP = episode of critical illness and intensive care unit treatment > 3 days within last 12 months
5. SSC–CIP is confirmed by ERC (if the first ERC is performed at baseline, the patient may be considered as screening failure if the diagnosis is not confirmed (see below))
6. Elevation of bilirubin ≥ 2.5 upper limit of normal (ULN) at screening
7. Elevation of alkaline phosphatase (AP) or gamma-glutamyl-transferase (GGT) > 2.5 ULN or elevation of both at screening
8. Women without childbearing potential defined as follows:

- At least 6 weeks after surgical sterilization by bilateral tubal ligation or bilateral oophorectomy or
- Hysterectomy or uterine agenesis or
- ≥ 50 years and in postmenopausal state > 1 year or
- < 50 years and in postmenopausal state > 1 year with serum follicle-stimulating hormone (FSH) > 40 IU/l and serum estrogen < 30 ng/l or a negative estrogen test, both at screening

or

Women of childbearing potential:

- Who are practicing sexual abstinence (periodic abstinence and withdrawal are not acceptable) or
- Who have sexual relationships with female partners only and/or with sterile male partners or
- Who are sexually active with fertile male partner, have a negative pregnancy test during screening, and agree to use reliable methods of contraception (failure rate of < 1% per year) from the time of screening until end of the clinical trial.

Patients are excluded from participation in the trial if they meet one of the exclusion criteria:

1. Patient is too unstable to undergo ERC
2. Inclusion in any other intervention trial within the last 30 days
3. Pregnancy or lactation period

#### Who will take informed consent

Patients (conscious or unconscious) with suspected SSC–CIP are considered for inclusion in this trial. The investigator is responsible for obtaining patient’s/legal representative’s written informed consent after adequate explanation of the aim, study assessments, potential risks and benefits, and consequences of the study as well as alternative treatment options. If patients are incapable of giving consent due to unconsciousness, informed consent may be given by a legal representative who has been designated by the local court. After retrieval of capacity for informed consent, patients have to be informed about study-specific interventions that have already been done and about the treatments that are planned in the future. Patients have to be asked if they want to continue participation in the trial and have to sign an informed consent form themselves. The patient information/informed consent form has to be signed in duplicate by the patient/legal representative and the investigator. One document will be given to the patient/legal representative; the other one remains in the trial investigator file (TIF) at the trial site. No study procedures are allowed to be conducted until patient’s/legal representative written informed consent has been obtained. The patient information/informed consent form has to be revised whenever important new information becomes available that may be relevant to the subject’s consent. The patients have to be informed and asked to give their consent to continue study participation by signing the updated form. Participation in this clinical trial is voluntary. Withdrawal from the trial at any time and for any reason is without any disadvantages to the patient’s further treatment.

#### Interventions

Patients with SSC–CIP are randomized either to the interventional or control arm. Patients in the experimental group will undergo scheduled invasive evaluation of the biliary tract with ERC and endoscopic interventions every 8 weeks until 6 months (24 weeks). Patients in the SOC group will receive only non-interventional standard of care treatment. Patients in both arms will be followed up every 8 weeks with clinical and laboratory assessment (Table 2). After 6 months (24 weeks), both groups are followed up every 8 weeks until 12 months.

Endoscopic evaluation has to be performed at baseline in both groups and during each ERC in the intervention group. Furthermore, an assessment of the biliary tract has to be made in each patient during V1 after diagnosis of SSC and before randomization to intervention group or control arm. Contrast injection with ERC catheter after attempt of bile extraction will be used in both groups. In the control arm, the procedure is terminated after contrast injection. They will be managed using a strictly conservative strategy that avoids biliary interventions unless such intervention is unequivocally mandated by the clinical situation. We foresee that this will be the case only in cholangiosepsis with obstruction which is part of the composite endpoint. Patients in the control arm will be seen every 8 weeks and a blood collection with acquisition of clinical data will be performed. After 6 months, a control MRCP will be performed in both groups in order to evaluate and compare the extent of bile duct damage between the groups. In the intervention group potential endoscopic interventions are as follows: sphincterotomy (V1-4), occlusion cholangiogram (V1-V4), extraction of all removable biliary casts via balloon, brush, basket and/or flushing (V1-V4), dilatation or bougienage of all endoscopically treatable bile duct stenoses (V1-V4), largest size of balloon or bougienage in relation to bile duct size has to be applied (V1-V4). Selective intubation of ductus hepaticus sinister and dexter with visualization and balloon cleaning is preferred (V1-V4). At the end of the procedure, flushing of the left and right biliary system with 20 ml Ringer’s lactate solution in order to flush remnants of contrast medium (V1-V4) and stent placement at physician’s assessment V1-V4 (high-grade stenosis of the bile ducts with insufficient dilatation or bougienage) will be performed. After the ERC, the following items have to be documented: procedure time, the extracted material (stones, cast, sludge and/or pus), largest size of balloon or bougienage if used, the possibly applied stent(s) and the potential use of intra-biliary antibiotics, flushing catheters or nasobiliary drainage (V1-4).

A bile specimen of up to 5 ml will be collected at baseline and visit 2 to visit 4 (only intervention-group) to analyze the microbial microenvironment in the biliary system and bile to gain insights in its role during SSC–CIP. Moreover, blood samples per visit of approx. 15 ml (serum/plasma) and 2.6 ml (genetic analysis, only screening) will be obtained for the evaluation of the exploratory endpoints.

#### Outcomes

##### Primary endpoint

The primary endpoint (Table 1) is the failure rate defined as a composite endpoint consisting of:

1. Occurrence of death or
2. Necessity of liver transplantation or
3. Occurrence of cholangiosepsis (defined by SEPSIS-3 criteria and diagnosis of acute cholangitis according to the Tokyo Guidelines), whatever occurs first

**Table 1:** Endpoints

**Primary endpoint**	Composite endpoint of: Cholangiosepsis (acute cholangitis (Tokyo criteria) and sepsis (Sepsis-3 criteria) Liver transplantation Death (whatever occurs first)
**Secondary endpoints**	
Single components of the primary endpoint	Cholangiosepsis Liver transplantation Death
Laboratory parameters	Bilirubin, alkaline phosphatase (AP), gamma-glutamyl transferase (GGT), aspartate aminotransferase (AST), alanine aminotransferase (ALT), lactate dehydrogenase (LDH), glutamate dehydrogenase (GLDH), creatinine, C-reactive protein (CRP), cholinesterase (CHE) as change from baseline
Model for end-stage liver disease score (MELD)	MELD as change from baseline
Unplanned hospital admissions	Necessity and days free of hospital care within 6 months
Unplanned intensive care unit (ICU) admissions	Necessity and days free of intensive care unit care, invasive ventilation, renal replacement therapy, vasopressors within 6 months
Necessity of anti-infective treatment	
**Safety endpoints**	
Death	
Liver transplantation	
Cholangiosepsis	
Cholangiosepsis-associated septic shock	
Acute on chronic liver failure (ACLF)	ACLF of any grade
Unexpected in-patient hospitalization or prolongation of existing hospitalization	
Unexpected ICU hospitalization	
ERC-related SAEs	Acute post ERC cholangiosepsis, acute post ERC pancreatitis, post ERC bleeding originating from the biliary system, duodenal or biliary perforation, aspiration pneumonia, allergic reaction to sedation associated with endoscopic procedure
Other SAEs	Other potentially life-threatening events as assessed by the individual investigator
**Exploratory endpoints**	
Biliary microbiome	Specific signatures of the biliary microbiome from biliary aspirate
Extent of bile duct damage	Extent of bile duct damage at 6-month MRCP compared to baseline as determined by central radiology reading

Survival is part of the primary endpoint because it is the most relevant endpoint and SSC–CIP is associated with significant mortality [2–4, 7, 9]. Liver transplantation is included as it is one treatment option that can restore liver function and avert death in advanced SSC–CIP functionally curing the disease [10]. Cholangiosepsis as systemic complication of cholangitis is a main trigger in the progress of the disease. Cholangiosepsis is promoted by bile duct stenoses which lead to deterioration of normal bile flow. Biliary complications and cholangiosepsis are frequent observations in SSC [6, 9]. In PSC, it has been shown that regular/scheduled endoscopic therapy of bile duct stenoses may lead to a reduction of cholangitis/cholangiosepsis and a prolonged transplant-free survival [11].

Cholangiosepsis can clearly be defined by SEPIS-3-criteria [12] and diagnosis of acute cholangitis according to the Tokyo Guidelines [13].

The SEPSIS-3 criteria validate sepsis based on an increase in the Sequential [Sepsis-related] Organ Failure Assessment (SOFA) score of 2 points or more, which is associated with an in-hospital mortality greater than 10%. The SOFA score [14] is obtained by assessing the following items:

- Respiration: PaO_2_/FiO_2_ (mmHg or kPa)
- Coagulation: platelets (× 10.3/μl)
- Liver: bilirubin (mg/dl or μmol/l)
- Cardiovascular: mean arterial pressure (MAP) and necessity of inotropes and/or vasopressors
- Central nervous system: Glasgow Coma Scale (GCS) [15]
- Renal: creatinine (mg/dl or μmol/l)

Because of preexisting organ dysfunction in the setting of secondary sclerosing cholangitis, the SOFA score will be assessed based on an increase of the SOFA score from baseline or the last study visit according to the SEPSIS-3 criteria.

Acute cholangitis is diagnosed according to the criteria of the 2018 Tokyo Guideline. For this purpose, signs of systemic inflammation, signs of existing cholestasis, and characteristic imaging are added. The diagnosis is considered confirmed when all criteria are met [13]. In detail, the following parameters are taken into account:

- (A) Systemic inflammation: fever and/or chills, abnormal white blood cell counts, increase of serum C-reactive protein levels, and other changes indicating inflammation
- (B) Cholestasis: jaundice, increased serum AP, GGT, AST, and ALT levels
- (C) Imaging findings: biliary dilatation, evidence of the etiology on imaging (stricture, stone, stent, etc.)

The diagnosis of acute cholangitis is definite in the presence of one item in A, one item in B, and one item in C. The diagnosis of acute cholangitis is suspected in presence of one item in A + one item in either B or C.

We therefore use the following definition of cholangiosepsis: cholangiosepsis = both parameters present:

- (1) Increase in SOFA score ≥ 2 points from baseline or last regular study-visit following SEPSIS 3 criteria.
  AND
- (2) Diagnosis of acute cholangitis according to the Tokyo Guidelines as seen above.

##### Secondary endpoints

The following secondary endpoints will be analyzed (Table 1):

1. Single components of the primary endpoint
2. Laboratory parameters as change from baseline
3. Model for end-stage liver disease score (MELD) as change from baseline
4. Unplanned hospital admissions
5. Unplanned Intensive care unit (ICU) admissions
6. Necessity of anti-infective treatment

##### Safety endpoints

A serious adverse event (SAE) is defined as any event that is potentially life threatening and may or may not be associated with the procedure under investigation. In detail, all following events are categorized as SAEs (Table 1):

- Death
- Liver transplantation
- Cholangiosepsis
- Cholangiosepsis-associated septic shock
- Acute on chronic liver failure (ACLF) of any grade
- Unexpected in-patient hospitalization or prolongation of existing hospitalization
- Unexpected ICU hospitalization
- ERC related SAEs
- Other potentially life-threatening events as assessed by the individual investigator

SAEs will be collected throughout the study and documented in the eCRF and on an SAE-form. The assessment of possible serious adverse events takes place by the investigator from inclusion of subjects into the study until the end of follow-up. SAEs including a risk–benefit evaluation of the study by the responsible investigator will be reported periodically (every 3 months) to the Ethics Committee according to the Declaration of Helsinki.

Given the profound severity of disease in the patients under investigation (hospitalized patients, mostly under critical care, multiple clinical, and laboratory abnormalities), adverse events (AEs), not fulfilling the definition of a SAEs (see above), will not be reported in this study.

#### Exploratory endpoints

As exploratory endpoints, specific signatures in the biliary microbiome and the extent of bile duct damage at 6-month MRCP compared to baseline (as determined by central radiology reading) will be analyzed.

#### Participant timeline

A time schedule of enrolment, interventions, assessments, and visits (SPIRIT figure) for participants is given as a schematic table (Table 2).

**Table 2:** Schedule of enrollment, interventions and assessments (SPIRIT figure)

Study phase	Screening	Baseline^a^	Treatment			Follow-up
**Visit procedures**	**SCR** Day − 28 to day 0	**V1** Day 1 (diagnosis SSC)	**V2** Week 8 ± 5 days	**V3** Week 16 ± 5 days	**V4/UN/ET** Week 24 (end of treatment) ± 5 days	**FU1-FU3** Every 8 weeks ± 5 days
**Clinical assessment**						
Informed consent	x					
Eligibility criteria	x					
Gender and age	x					
Medical history	x					
ICU related history	x					
Vital signs and oxygen saturation	x	x	x	x	x	x
Height and skin color	x					
Weight	x	x	x	x	x	x
Concomitant medication	x	x	x	x	x	x
Pregnancy prevention counseling	x	x	x	x	x	
Endoscopic evaluation (ERC)		x	x (intervention group)	x (intervention group)	x (intervention group or clinically necessary for UN/ET)	
MRCP^b^	x				x	
Endoscopic intervention (ERC)		x (endoscopic evaluation only at non-intervention group)	x (intervention group)	x (intervention group)	x (intervention group or clinically necessary for UN/ET)	
Randomization		x				
Checking the components of the primary endpoint^c^			x	x	x	x
Secondary endpoints^d^					x	
SOFA score		x	x	x	x	x
Serious adverse events		x	x	x	x	x
**Laboratory assessment**						
Hematology	x	x	x	x	x	x
Serum chemistry	x	x	x	x	x	x
Thyroid function test	x					
Serum or urine ß-hCG pregnancy testing^e^	x	x	x	x	x	
Genetic sample	x					
Serum/plasma	x	x	x	x	x	x
Bile specimen		x	x (intervention group)	x (intervention group)	x (intervention group or clinically necessary for UN/ET)	

^a^Baseline/day 1 assessments must be performed prior to treatment

^b^MRCP at screening if possible. MRCP at visit 4 may only be performed if MRCP has been conducted during screening (not for unscheduled visit (UN), preferably prior to ERC)

^c^The primary endpoint is defined as occurrence of death or necessity of liver transplantation or occurrence of cholangiosepsis, whatever occurs first

^d^Secondary endpoints: single components of the primary endpoint: liver transplantation, death, cholangiosepsis; Laboratory parameters (bilirubin, AP, GGT, AST, ALT, LDH, GLDH, creatinine, CRP, CHE) as change from baseline; model for end-stage liver disease score (MELD) score as change from baseline; unplanned hospital admissions (necessity and days free of hospital care within 6 months); unplanned intensive care unit (ICU) admissions (necessity and days free of intensive care unit care, invasive ventilation, renal replacement therapy, vasopressors within 6 months), necessity of anti-infective treatment

^e^Serum pregnancy testing at screening and urine pregnancy testing during the study for females of childbearing potential only (if urine pregnancy testing is positive, a confirmation with serum pregnancy testing is required)

#### Sample size

The primary aim of the study is to demonstrate superiority of the experimental group compared to the control group in patients with SSC–CIP. The primary endpoint is a failure rate defined as a composite endpoint including liver transplantation, death, or cholangiosepsis after 6 months. Sample size calculation was conducted using a two-group continuity corrected chi-square test in nQuery Advisor 7.0. The type I error is set to 5% (two-sided) and the study aims for a power of 80%. In the present trial, a rate of 70% regarding the composite endpoint is expected in the control group. This assumption is based on the following observations: prognosis of SSC–CIP is dismal with an estimated survival of less than 50% of patients who develop SSC–CIP within 3–6 months [2, 4, 7]. The transplant-free survival of SSC–CIP differs between publications. However, in our large cohort, transplant-free survival was 38.9% at 6 months [9, 16]. Additionally, biliary complications in SSC–CIP are numerous affecting up to 90% of patients [6]. Cholangiosepsis accounts for 20–33% of these biliary complications [6]. A total reduction of 30% is assumed for sample size calculation. This number is estimated from clinical observation and experience of patients with SSC–CIP and PSC [11]. To demonstrate a reduction of the rate from 70 to 40% in the experimental group, a sample size of 49 patients per group is needed leading to *n* = 98 in total. The expected drop-out rate is assumed to be 5% (estimation from clinical practice), which leads to a sample size of 52 patients per study arm and 104 patients in total.

#### Recruitment

SSC–CIP is characterized by a grim prognosis without established diagnostic or therapeutic algorithms. From the clinical experience of the investigators, patients and their legal representatives are highly motivated and show a strong adherence to therapy and schedules due to the severity of the disease. As liver transplantation is the only validated treatment option for these patients’ referral to and treatment in tertiary care centers is of main importance [8], therefore, we expect that the number of loss to follow-up is negligible. All attempts will be undertaken to collect the information about the primary endpoint for patients that did drop-out from the study or were withdrawn. Informed consent will already address the option to contact the patient for follow-up investigations after termination of the observational period of the initial clinical trial. Nevertheless, patients where the information on the primary endpoint is not available at month 6 will be counted as treatment failures.

#### Assignment of interventions: allocation

As screening tool, patients that are suspected to have SSC–CIP by medical history, laboratory results, and sonography will undergo non-invasive imaging by magnetic resonance cholangiopancreatography (MRCP) first (Table 2). Bile duct changes in SSC–CIP will be assessed by means of MRCP at screening and visit 4. Completion of MRCP requires that all of the inclusion criteria and none of the exclusion criteria are fulfilled and that the patient has no contraindications to undergo magnetic resonance imaging (MRI). MRCP at visit 4 may only be performed if MRCP has been conducted during screening. As there are currently no technical specifications on how to perform MRCP in SSC, acquisition parameters and quality assurance should be according to existing recommendations for patients with primary sclerosing cholangitis (PSC) [17, 18]. Datasets will be sent to the Study Center in the Department of Diagnostic and Interventional Radiology of Hannover Medical School for central reading and quality assurance. As there are currently no reporting standards for SSC, image interpretation and reporting will be based on recommendations for patients with PSC [19].

Only if MRCP confirms morphologic biliary changes consistent with a high suspicion for SSC–CIP, patients will undergo consequent diagnostic ERC evaluation. Randomization will be made following definitive diagnosis of SSC–CIP via diagnostic ERC. After cannulation of the biliary system with a small ERC catheter (5F, 1.7 mm diameter), the contrast agent will be injected, and in case of typical findings of SSC (irregular bile duct system with contrast filling defects, stenoses, and prestenotic bile duct dilation), patients will be randomized. In patients randomized in the control arm, no further intervention will be performed at this point.

Permuted block randomization with variable block length stratified by center will be used to allocate patients to both study arms. Only the statistician and the unblinded data manager will have access to the randomization list. The allocation is performed via the eCRF system. Only after the patient has been documented with inclusion and exclusion criteria in the eCRF by the study site, the allocation of the patient to a treatment group will be displayed in the eCRF automatically according to the sequence determined by the randomization list. For the study sites, it is not possible to see the randomization list.

#### Data collection and management

All study data will be collected by the investigator and/or other study personnel. A clinical trial data base is provided, in which the data are entered via an eCRF. Authorized and trained staff of the study sites will enter the data in the eCRF. SAEs will additionally be documented electronically. Verification of the data in the eCRF occurs by monitoring as well as via range, validity, and consistency checks programmed in the system. Additionally, manual queries can be raised in the system by authorized study staff if further discrepancies are detected. Based on the queries, the investigator can review and answer the found discrepancies directly in the system. All changes of data entered in the eCRF can be followed by an audit trail. A quality control will be performed before the database is closed. This procedure is documented. Finally, data transfer takes place for statistical evaluation. The data management plan contains further details about data management processes.

All study staff has to give due consideration to data protection and medical confidentiality. The collection, transfer, storage, and analysis of personal study-related data are performed pseudonymized according to national regulations. The declaration of data protection is contained within the patient information/informed consent form.

An independent data safety monitoring board (DSMB) will be implemented to detect possible harms and to assure continuous risk/benefit assessment. The DSMB is a group of independent experts external to the clinical investigation assessing the progress, safety data, and, if needed, critical efficacy endpoints. Details of the definition of DSMB, its composition, and its roles and responsibilities will be set forth in a separate DSMB charter.

#### Collection and storage of biomaterial

A bile specimen of up to 5 ml will be collected at baseline and visit 2 to visit 4 (only intervention-group) to analyze the microbial microenvironment in the biliary system and bile to gain insights in its role during SSC–CIP. Moreover, blood samples per visit of approx. 15 ml (serum/plasma) and 2.6 ml (genetic analysis, only Screening) will be obtained for the evaluation of further exploratory endpoints. Details regarding the collection, processing, storage, and shipment of samples will be included in an additional lab manual. In short, specimens will be stored at − 80° C and will be shipped for central analysis to Hannover Medical School. Additional written informed consent of patients or relatives will be obtained prior to collecting biomaterial.

### Statistical methods

#### Analysis of the primary endpoint

The primary aim of the study is to demonstrate that programmed endoscopic therapy (experimental group) compared to a conservative strategy (control group) reduces the occurrence of treatment failures defined as death or necessity of liver transplantation or development of cholangiosepsis 6 months after randomization.

The primary analysis will be performed according the intention-to-treat (ITT) principle. Specifically, all patients will be analyzed as randomized. The occurrence of the primary endpoint until month 6 will be analyzed by a stratified Mantel–Haenszel estimate for the risk difference (experimental minus control group) with center as stratification factor. If the upper bound of the corresponding 95% confidence interval is below 0, superiority of the experimental group will be concluded. Missing values for the primary endpoint will be counted as treatment failures. Sensitivity analyses will be carried out in the per-protocol population, which consists of all patients who complete the study in accordance to the protocol. According to this, the primary estimand is defined as described in Table 3.

**Table 3:** Analysis of the primary endpoint

**Objective:** Demonstrate superiority of programmed endoscopic therapy compared to standard of care on the composite endpoint of all-cause mortality, liver transplantation and cholangiosepsis 6 months after randomization	
**Target population:** Adult patients with by ERC confirmed SSC–CIP	
**Variable/endpoint:** Occurrence of any of the events	
**Primary estimand:**	
Intercurrent events	Early discontinuation of the study
Strategy	Treatment policy
Population-level summary	A Mantel–Haenszel estimate for the risk difference (experimental minus control group) with center as stratification factor will be used to compare treatment failures in all randomized patients
Estimand description	Superiority of the experimental group on the composite endpoint of death, liver transplantation or cholangiosepsis will be measured using the Mantel–Haenszel estimate for the risk difference irrespectively from the early discontinuation of the study
Imputation/data/censoring rule(s)	Patients who early discontinue the study without assessment of the primary endpoint will be counted as treatment failures
Sensitivity analyses	1)Estimation of the risk difference and the corresponding 95% CI without adjustment for center 2)Estimation of the risk difference and the corresponding 95% CI in a model where patients who early discontinue the study without assessment of the primary endpoint are counted as treatment responders 3)Estimation of the risk difference and the corresponding 95% CI in the per-protocol population

#### Analysis of secondary endpoints

All secondary analyses will be exploratory and will be conducted on the ITT population and will be stratified by center.

The single components of the composite primary endpoint will be analyzed in line with the primary analysis using a Mantel–Haenszel estimate for the risk difference.

Necessity of antibiotic treatment, unplanned hospital admission, and ICU admission will be analyzed using a Mantel–Haenszel estimate for the risk difference.

For time to primary endpoint and time to components of the primary endpoint, a Cox regression will be conducted with treatment group and center as independent variables.

The laboratory parameters (bilirubin, alkaline phosphatase, gamma-glutamyltransferase, aspartate aminotransferase, alanine aminotransferase, lactate dehydrogenase, glutamate dehydrogenase, creatinine, albumin, c-reactive protein, cholinesterase, estimated glomerular filtration rate (eGFR) (CKD-EPI formula), and MELD) are assessed with an analysis of covariance (ANCOVA) with change from baseline as dependent variable and treatment, baseline, and center as independent variables.

#### Interim analyses

No interim analyses will be performed.

#### Analysis of safety endpoints

Occurrence of complications and adverse and serious adverse events will be analyzed descriptively using absolute and relative frequencies for the whole population and separately for the experimental and control groups and will be compared with chi-squared tests.

#### Analyses populations

The primary analysis will be conducted on the ITT-population including all patients that have been randomized. Patients will be analyzed as randomized independently of the performed intervention.

The per-protocol (PP)-population comprises all patients that received the randomized group and were complying with the study-protocol until the end of the observational period and particularly remained in the treatment arm they were allocated to by randomization.

Safety population: In the safety population, all patients are analyzed who are randomized. Patients will be analyzed as treated.

### Oversight and monitoring

#### Responsibilities

This study will be conducted in compliance with the ICH GCP guidelines (as far as possible for this kind of study) and the Declaration of Helsinki. Investigators must have sufficient time to conduct the clinical study in compliance with the study protocol. Furthermore, they have to accurately and completely enter study data in the eCRF. Investigators are responsible for obtaining informed consent of the patients as well as for the preparation and maintenance of adequate case files in order to record observations and other data relevant for this clinical study. Besides, they have to file the study-related records in the ISF and have to maintain its actuality. They will permit study-related monitoring visits. The investigator must provide direct access to the study site’s facilities, to source documents, and to all other study documents.

#### Favorable opinion of independent ethics committees

A favorable opinion of the lead Independent Ethics Committee at Hannover Medical School (Ethikkommission Medizinische Hochschule Hannover) has been obtained prior to study initiation (No 10237_BO_S_2022) in March 2022. Additionally, a favorable opinion will be obtained from each of the center specific Ethics Committee prior to initiation of each site. Written, informed consent to participate will be obtained from all participants.

#### Monitoring

Monitoring is performed for reasons of quality assurance and to verify that the study is conducted according to the protocol as well as to legal and regulatory requirements applicable for clinical trials, particularly the International Conference on Harmonisation (ICH) GCP guidelines.

Quality assurance is based on three components: on-site monitoring, central monitoring, and extensive training. All trial-related processes will follow the SOPs of the Zentrum für Klinische Studien (ZKS) of Hannover Medical School. Central monitoring will include a timely query management process based on consistency and plausibility checks automatically generated from the database, combined with a reminder process for missing documentation. A monitoring plan serves as a guiding document and describes quality assurance details including monitoring activities, responsibilities, and processes. The project manager and/or clinical research associate(s) (CRA(s)) prepares the monitoring plan and reconciles with the coordinating investigator and members of the (clinical) project team.

Pre-study visits/video conferences will be performed in each recruiting study site by independent ZKS CRA to initially check on feasibility of the planned project and to clarify study-specific issues. Site initiation visit/video conferences have to be performed in order to instruct the local investigators in the study protocol, further essential study documents, and documentation of data. Monitoring will be performed by CRAs of the ZKS and/or by subcontracted, qualified, and trained freelancer(s) on a regular basis dependent on specific requirements of the individual study centers. During the study, the CRA will visit the investigational site periodically to check the completeness of subject records, accuracy of entries in the eCRF, and adherence to the protocol and to ICH-GCP. Source data verification will be done according to a risk adapted approach to assure high data quality and patient safety. The focus of on-site monitoring will be on the verification of informed consent documents, eligibility criteria, primary endpoint, key secondary endpoints, and safety aspects. Close-out visits will be done at the end of the trial and in case a site will prematurely be closed.

Key study site personnel must be available to assist the CRA during monitoring visits. The investigator must give the CRA access to all relevant source documents to confirm their consistency with the CRF entries. The investigator must maintain source documents for each subject in the study, consisting of case and visit notes containing demographic and medical information, laboratory data, and the results of any other tests or assessments. All entered information in eCRFs must be traceable to these source documents in the subject’s file. Data not requiring a written or electronic record will be defined before study start and will be recorded directly in the eCRFs which will be documented as being the source data. The investigator must also keep the original of the signed informed consent form(s).

#### Record retention

All relevant study-related documents have to be archived for at least 10 years after completion or premature discontinuation of the clinical study. The investigator agrees to keep the ISF, including the identity of all participating patients, all original signed informed consent forms, detailed records of treatment, and all other applicable study-related documents as well as source documents. The records should be retained by the investigator for at least 10 years after completion or premature discontinuation of the clinical study. Source data have to be kept according to national regulations.

#### Insurance

The trial will be covered by a participant insurance in case the trial site (clinic) does not cover the study by its liability insurance (Haftpflichtversicherung). All subjects/legal representatives will be informed about their rights and obligations in regard to insurance policies before participating in the study. A copy of the insurance policies will be handed out to each patient/legal representative.

#### Financing

This study is funded by the German Research Foundation (DFG, VO 2458/1–1).

#### Amendments

Each amendment of essential study documents has to be approved by the study initiators. Favorable opinion of IEC is required for amendments prior to implementation.

#### Dissemination plans

It is anticipated to publish the results of the clinical trial in a scientific medical journal and at national and international meetings. The responsible investigator will designate the first and the last authors of the publication. The order of subsequent authors will be allocated according to the number of patients recruited by each site. Data will be available to investigators upon reasonable request.

The trial was registered at ClinicalTrials.gov (NCT05396755, date of registration: May 31, 2022, last update: May 31, 2022).

## Discussion

To the present time, no adequately powered randomized clinical trial has investigated the effect of programmed ERC interventions on complications and survival in patients with SSC–CIP.

There is no evidence-based standard of care for patients with SSC–CIP. However, based on the phenotypical similarities to patients with PSC, it is believed that similar treatment strategies might be of benefit in SSC–CIP as well. Therapeutically comparable courses are seen in primary sclerosing cholangitis (PSC). Endoscopic treatment of higher-grade strictures provides clinical benefit in PSC with decreased symptoms of pruritus or cholangitis [20]; it has been further shown that regular/scheduled endoscopic therapy of biliary stenosis can lead to a reduction in cholangitis/cholangiosepsis and prolonged transplant-free survival [11].

In contrast, in SSC–CIP, these endpoints after endoscopic interventions, particularly the prolongation of transplant-free survival and reduction of cholangitis/cholangiosepsis, have not been investigated in prospective, randomized controlled studies yet. Endoscopic intervention has already been shown to be associated with lower bilirubin and ALP levels and short-term clinical improvement [2, 4].

On the other hand, if the present study provides negative results, the risk associated to ERCs including the use of radiation as well as the risk of ERC-related complications such as bleeding, cholangitis, and pancreatitis could be avoided in the future in patients with SSC–CIP. The alternative rationale for the conservative strategy without regular endoscopic intervention followed in the treatment of patients in the control arm of the study is to minimize bile duct manipulation to prevent increasing introduction of multidrug-resistant intestinal bacteria into the biliary system. It has been shown that drug- or multidrug-resistant bacteria and fungal infections are particular risk factors in the bile of SSC patients [21]. It is therefore reasonable to assume that endoscopic manipulation of the biliary tract system could further exacerbate a corresponding burden of non-physiologic microbiota in the bile duct system.

Therefore, the study data that will be collected in this study will most likely establish an evidence-based therapeutic strategy in patients with SSC–CIP concerning a potential benefit of regular biliary interventions.

The investigators are convinced that a risk–benefit consideration clearly favors performance of the here proposed clinical trial. Importantly, this is an investigator-initiated trial, funded by the DFG without any competing commercial or financial interests involved.

Furthermore, accompanying systematic and longitudinal biomaterial analysis will most certainly enable highly stimulating scientific investigations in the field of SSC–CIP related research.

## Conclusion

This trial has substantial clinical relevance as it prospectively evaluates for the first time a treatment option for patients with SSC–CIP. A positive trial result could change the current standard of care for SSC–CIP.

## Data Availability

Data will be available to investigators upon reasonable request.

## Abbreviations

AE: Adverse event
AP: Alkaline phosphatase
ARDS: Adult respiratory distress syndrome
BMI: Body mass index
CRA: Clinical research associate
DSMB: Data safety monitoring board
ECMO: Extracorporeal membrane oxygenation
eCRF: Electronic case report form
eGFR: Estimated glomerular filtration rate
ERC: Endoscopic retrograde cholangiography
ET: Early termination
FiO_2_: Fraction of inspired oxygen
FSH: Follicle-stimulating hormone
GCP: Good Clinical Practice
GCS: Glasgow Coma Scale
GGT: Gamma-glutamyl-transferase
IB: Investigator’s Brochure
ICH: International Conference on Harmonization
ICU: Intensive care unit
IEC: Independent Ethics Committee
ISF: Investigator Site File
ITT: Intention-to-treat
MAP: Mean arterial pressure
MRCP: Magnetic resonance cholangiopancreatography
MRI: Magnetic resonance imaging
PaO_2_: Partial pressure of oxygen
PD: Protocol deviations
PEEP: Positive end-expiratory pressure
PSC: Primary sclerosing cholangitis
SAE: Serious adverse event
SOFA: Sepsis-related organ failure assessment score
SOP: Standard operating procedure
SCR: Screening
SSC: Secondary sclerosing cholangitis
SSC–CIP: Secondary sclerosing cholangitis in critically ill patients
TIF: Trial investigator file
ULN: Upper limit of normal
UN: Unscheduled visit
CTS: Clinical trial services
